# ^1^H-MRS neurometabolite profiles and motor development in school-aged children who are HIV-exposed uninfected: a birth cohort study

**DOI:** 10.3389/fnins.2023.1251575

**Published:** 2023-10-12

**Authors:** Simone R. Williams, Frances C. Robertson, Catherine J. Wedderburn, Jessica E. Ringshaw, Layla Bradford, Charmaine N. Nyakonda, Nadia Hoffman, Shantanu H. Joshi, Heather J. Zar, Dan J. Stein, Kirsten A. Donald

**Affiliations:** ^1^Department of Paediatrics and Child Health, Red Cross War Memorial Children’s Hospital, University of Cape Town, Cape Town, South Africa; ^2^Neuroscience Institute, University of Cape Town, Cape Town, South Africa; ^3^Department of Human Biology, University of Cape Town, Cape Town, South Africa; ^4^Cape Universities Body Imaging Centre (CUBIC), Cape Town, South Africa; ^5^Department of Clinical Research, London School of Hygiene & Tropical Medicine, London, United Kingdom; ^6^Department of Psychiatry and Mental Health, University of Cape Town, Cape Town, South Africa; ^7^Departments of Neurology and Bioengineering, UCLA, University of California, Los Angeles, Los Angeles, CA, United States; ^8^SAMRC Unit on Child & Adolescent Health, University of Cape Town, Cape Town, South Africa; ^9^SAMRC Unit on Risk and Resilience in Mental Disorders, University of Cape Town, Cape Town, South Africa

**Keywords:** HIV exposure, brain development, magnetic resonance spectroscopy, neuroimaging, metabolites

## Abstract

**Objective:**

Alterations in regional neurometabolite levels as well as impaired neurodevelopmental outcomes have previously been observed in children who are HIV-exposed uninfected (CHEU). However, little is known about how neurometabolite profiles may relate to their developmental impairment. This study aimed to compare neurometabolite concentrations in school-aged CHEU and children who are HIV-unexposed (CHU) and to explore associations of neurometabolite profiles with functional neurodevelopment in the context of perinatal HIV exposure.

**Methods:**

We used 3 T single voxel proton magnetic resonance spectroscopy (^1^H-MRS) to quantify absolute and relative neurometabolites in the parietal gray and parietal white matter in school-aged CHEU and aged- and community-matched CHU. Functional neurodevelopmental outcomes were assessed using the early learning outcome measure (ELOM) tool at 6 years of age.

**Results:**

Our study included 152 school-aged children (50% males), 110 CHEU and 42 CHU, with an average age of 74 months at the neuroimaging visit. In an adjusted multiple linear regression analysis, significantly lower glutamate (Glu) concentrations were found in CHEU as compared to CHU in the parietal gray matter (absolute Glu, *p* = 0.046; Glu/total creatine (Cr+PCr) ratios, *p* = 0.035) and lower total choline to creatine ratios (GPC+PCh/Cr+PCr) in the parietal white matter (*p* = 0.039). Using factor analysis and adjusted logistic regression analysis, a parietal gray matter Glu and myo-inositol (Ins) dominated factor was associated with HIV exposure status in both unadjusted (OR 0.55, 95% CI 0.17–0.45, *p* = 0.013) and adjusted analyses (OR 0.59, 95% CI 0.35–0.94, *p* = 0.031). With Ins as one of the dominating metabolites, this neurometabolic factor was similar to that found at the age of two years. Furthermore, this factor was also found to be correlated with ELOM scores of gross motor development in CHEU (Pearson’s *r* = −0.48, *p* = 0.044). In addition, in CHEU, there was a significant association between Ins/Cr+PCr ratios in the parietal white matter and ELOM scores of fine motor coordination and visual motor integration in CHEU (Pearson’s *r* = 0.51, *p* = 0.032).

**Conclusion:**

Reduced Glu concentrations in the parietal gray matter may suggest regional alterations in excitatory glutamatergic transmission pathways in the context of perinatal HIV and/or antiretroviral therapy (ART) exposure, while reduced Cho ratios in the parietal white matter suggest regional myelin loss. Identified associations between neurometabolite profiles and gross and fine motor developmental outcomes in CHEU are suggestive of a neurometabolic mechanism that may underlie impaired motor neurodevelopmental outcomes observed in CHEU.

## 1. Introduction

Perinatal vertical HIV transmission is the common mode of HIV transmission in children. Intervention programs aimed at reducing the mother-to-child transmission of HIV have successfully led to a decreasing number of new perinatal HIV infections, resulting in a growing population of children who are HIV-exposed uninfected (CHEU) with 15.4 million CHEU reported globally in 2020 (Slogrove et al., 2020). CHEU are often exposed to HIV and/or antiretroviral therapy (ART) during perinatal brain development; therefore, it is important to understand the impact of perinatal HIV and/or ART exposure on early neurodevelopment in CHEU.

During early perinatal development, important cellular neurodevelopmental processes such as neurogenesis, neuronal migration, and synaptogenesis occur (Silbereis et al., 2016; Dehorter and Del Pino, 2020), which form the basis upon which optimal brain health and function are built (Hertzman and Wiens, 1996). Given that perinatal exposure to viral and bacterial agents can affect fetal brain development (al-Haddad et al., 2019), exposure to maternal HIV and/or ART during early neurodevelopment may adversely affect functional brain development and subsequent long-term neurological functioning. Brain developmental outcomes have previously been investigated in CHEU, with studies reporting significantly worse functional neurodevelopmental outcomes in areas of language and motor function in CHEU when compared to children who are HIV-unexposed (CHU) during the first 2 years of life (Wedderburn et al., 2022). In a South African birth cohort of 2-year-old CHEU, receptive and expressive language function was found to be delayed (Wedderburn et al., 2019). In addition, significantly delayed gross motor development in CHEU at the age of 6 months has been reported (le Roux et al., 2018). Significantly worse neurofunctional outcomes have also been reported in older CHEU. For example, a study in Zambia found 8-year-old CHEU to have poorer math scores when compared to CHU (Nicholson et al., 2015). Another study conducted in Thailand reported CHEU to have significantly lower IQ and memory scores at an average age of 7 years (Kerr et al., 2014). Although studies found poorer cognitive outcomes in school-aged CHEU, little is known about motor development at this age. Using magnetic resonance imaging (MRI) techniques as quantitative measures of brain development in CHEU, studies have reported significant neurostructural alterations, including reduced gray matter volumes, in infants who are HIV-exposed uninfected compared to CHU (Wedderburn, 2022) and compromised white matter integrity in infants (Tran et al., 2016) and older children (Jankiewicz et al., 2017).

Magnetic resonance spectroscopy (MRS) is a technique used to quantify specific metabolites in the brain by detecting the proton signals from metabolites within a given volume of interest (VOI) (Hajek and Dezortova, 2008). Using MRS, a study reported significant neurometabolite alterations in the basal ganglia in CHEU at the age of 9 years compared to matched CHU (Graham et al., 2020). In the same cohort of CHEU, similar changes in metabolite concentrations were found in the mid-frontal gray matter and peritrigonal white matter at the age of 11 years (Robertson et al., 2018). At both age points, reduced concentrations of N-acetyl aspartate (NAA) and Glutamate (Glu) were observed, suggesting that perinatal exposure to HIV and/or ART may affect regional neuronal integrity and glutamatergic pathways in the developing brain. Furthermore, a neurometabolic pattern identified through factor analysis was found to be a predictor of HIV exposure status in young 2-year-old (Bertran-Cobo et al., 2022) CHEU enrolled in the Drakenstein Child Health Study (DCHS). The neurometabolic pattern identified was dominated by myo-inositol (Ins) concentrations, a marker of inflammatory processes across parietal gray and white matter. Overall, regional neurometabolite patterns support findings from individual neurometabolite concentrations, providing a better understanding of how individual neurometabolites interact with each other within the context of perinatal HIV exposure.

Although regional neurometabolite concentrations and their association with functional neurodevelopmental outcomes have been investigated in the context of pediatric HIV infection (Keller et al., 2004), the link between regional neurometabolite profiles and functional neurodevelopmental outcomes in CHEU has not yet been explored. Metabolites in the brain are tightly regulated as metabolites mediate numerous metabolic and signaling pathways underlying various brain functions, including perception (Kandel et al., 2000), motor control (Kandel et al., 2000), homeostasis (Boison et al., 2017), and cognition (Nichols and Newsome, 1999). Therefore, alterations in neurometabolite concentrations in the context of perinatal HIV exposure could be involved in the pathophysiological mechanism underlying impaired functional neurodevelopmental symptomology in CHEU. To this end, we compared MRS-quantified neurometabolite concentrations between school-aged CHEU and CHU enrolled in the DCHS, building on prior findings at 2 years of age, and explored the association between regional neurometabolite profiles and functional neurodevelopmental outcomes.

## 2. Methods

### 2.1. Study design

This study was performed as a nested sub-study, recruiting pediatric participants enrolled in the Drakenstein Child Health Study (DCHS). The DCHS is a multidisciplinary longitudinal birth cohort study located in the peri-urban Drakenstein district, in Paarl, Western Cape, South Africa, and is an ongoing observational study aimed at investigating how early exposure to environmental, infectious, nutritional genetic, psychosocial, maternal, and immunological risk factors affect various aspects of child health and development (Stein et al., 2015; Zar et al., 2015; Donald et al., 2018, 2019).

### 2.2. Participants

Between March 2012 and March 2015, pregnant women attending routine antenatal care at two health clinics, Mbekweni and TC Newman, were recruited into the DCHS, with 1,143 live infants enrolled in the study at baseline and followed longitudinally (Donald et al., 2018). Children enrolled in the DCHS were invited to part take in a longitudinal neuroimaging sub-study, where participants received an MRI scan as neonates (aged 2–6 weeks), young children (aged 2–3 years), and school-aged children (aged 6–7 years). Functional neurodevelopmental assessments were also administered longitudinally to children enrolled in the DCHS at timepoints ranging from 6 months to 6 years of age. Neuroimaging data and functional neurodevelopmental outcomes assessed at the 6–7 year timepoint formed the basis of our analysis. Participants were included in the embedded neuroimaging study if they had none of the following exclusion criteria: any pre-existing medical conditions (HIV infection, neurological disorders, severe congenital malformations, chromosomal abnormalities, or genetic disorders) or a maternal history of illicit drug use during pregnancy. Ethical approval was obtained from the Faculty of Health Science, Human Research Ethics Committee at the University of Cape Town (HREC REF:438/2021, 044/2017, 525/2012) and by the Western Cape Provincial Health Research Committee (REF: 2011RP45). Written consent and assent were obtained from the parents or guardians and children over the age of 5 years, respectively.

### 2.3. Sociodemographic data collection

All sociodemographic and maternal psychosocial data were collected between 28 and 32 weeks of gestation though interviews and questionnaires described elsewhere (Zar et al., 2015; Donald et al., 2018, 2019). Infant birth anthropometry data were collected at delivery, while data on infant feeding methods and exclusive breastfeeding duration were obtained at ages 6–14 weeks, 6 months and 9 months. Maternal alcohol use during pregnancy was assessed using the Alcohol, Smoking, and Substance Involvement Screening Test (ASSIST), and maternal depression during pregnancy was assessed using the Edinburgh Postnatal Depression Scale. CHEU is defined as children born to HIV-infected mothers but are not HIV-infected. Maternal HIV status was confirmed via routine tests during pregnancy, with results confirmed every 12 weeks. All HIV-exposed uninfected children received HIV testing and were confirmed to have a negative HIV test result at approximately 18 months of age or once the mother had stopped breastfeeding if this lasted longer than 18 months. According to the prevention of mother-to-child transmission guidelines at the time, mothers living with HIV who were not already on triple first-line antiretroviral therapy (ART) initiated ART regimens [consisting of two nucleoside reverse transcriptase inhibitors and a non-nucleoside reverse transcriptase inhibitor, with the common combination being Tenofovir, Emtricitabine and Efavirenz (EFV)]. All HIV-exposed children received infant prophylaxis with either AZT or nevirapine for 6 weeks after birth without breastfeeding. Breastfed children received nevirapine throughout the breastfeeding period and for 1 week thereafter. Data on maternal CD4 cell count and viral load during pregnancy were extracted from clinical records and the online National Health Laboratory Service System.

### 2.4. MRS data collection and processing

**Figure 1:**
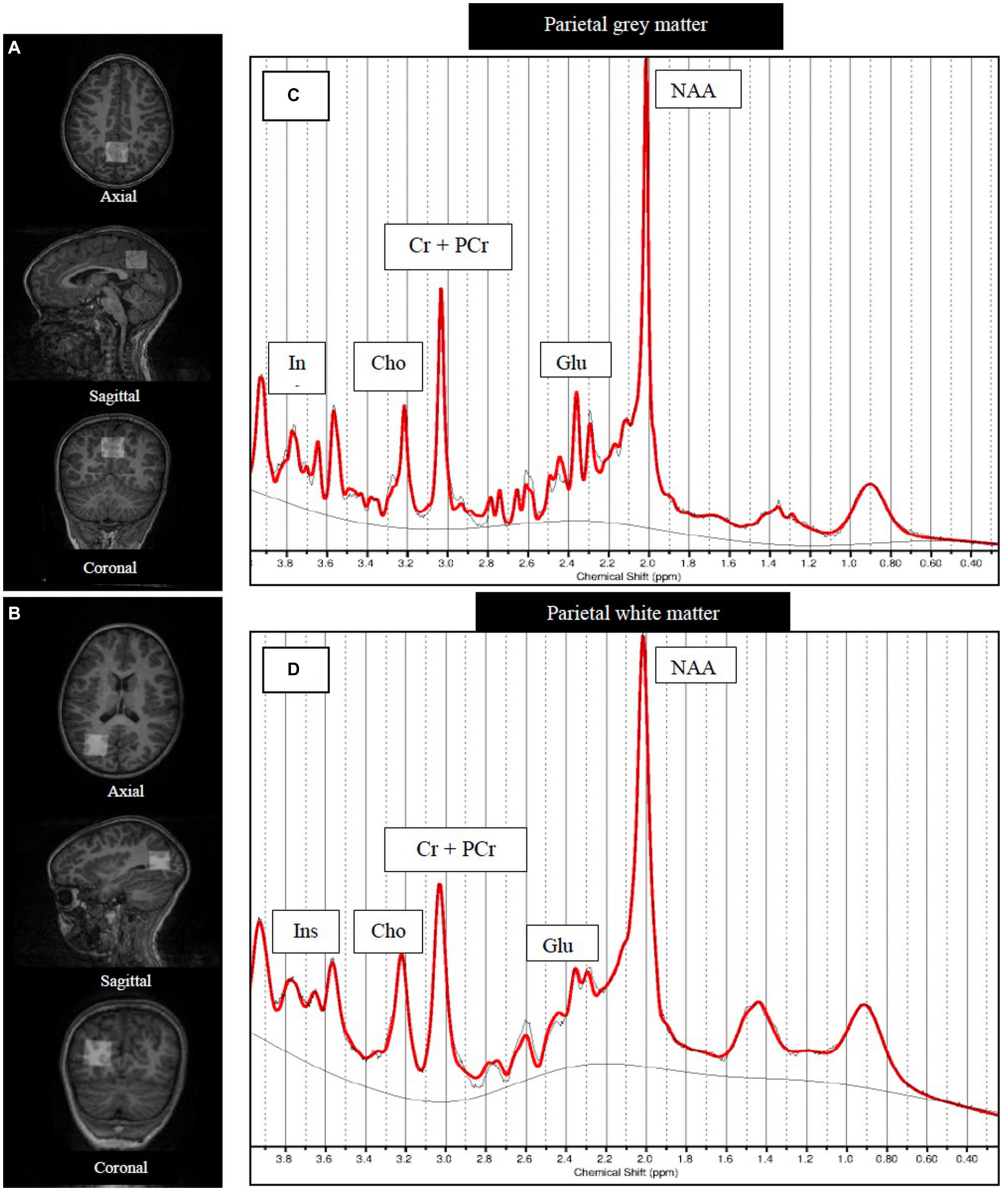
Structural MRI images showing MRS voxel placements and example SVS spectra [LCModel output]. Axial, sagittal and coronal views of the single voxel (25 × 25 × 25 mm3) placed covering **(A)** parietal gray matter and **(B)** right hemisphere parietal white matter. Examples of **(C)** parietal gray matter and **(D)** parietal white matter MRS spectra. NAA, N-acetyl aspartate; Glu, Glutamate; GPC+PCh, total choline (glycerophosphocholine +phosphocholine); Cr+PCr, total creatine (creatine +phosphocreatine).

Using a 32-channel head coil and without sedation, MRI data were acquired on a 3 T Skyra (VE 11) MRI scanner (Siemens, Erlangen, Germany) at the Cape Universities Body Imaging Center (CUBIC) in Cape Town, Western Cape. Upon arrival, children and their parents or guardians were directed to the child-friendly area at CUBIC, where the details of the MRI visit were shared, informed consent and assent were obtained, an introductory video on MRI scanning was offered to help parents and children understand the process of the MRI visit, and an MRI safety screening questionnaire was administered. After the consultation process, the children were given the opportunity to pick a movie of their choice to watch during the MRI scan. For MRS voxel positioning, high-resolution T1-weighted images were acquired using a magnetization prepared rapid gradient echo (MPRAGE) acquisition (FOV 256 × 256 mm^2^, TR 2500 ms, T1 1,000 ms, TE 3.35 ms, bandwidth 240 Hz/Px, resolution 1 × 1 × 1 mm^3^). Single ^1^H-MRS voxels (25 × 25 × 25 mm^3^) were placed covering parietal gray matter and parietal white matter regions (Figure 1). MRS data were acquired using a point resolved spectroscopy (PRESS) acquisition (TE 30 ms, TR 2,000 ms, 128 averages, bandwidth 1,200 Hz, and vector size 1,024) (Hess et al., 2011), with chemical shift selective (CHESS) water suppression (Haase et al., 1985). Single MRS scans without CHESS suppression were acquired to serve as water references. Automatic shimming was performed over voxel volumes through advanced scanner adjustments, with manual shimming performed where necessary, to reduce spectral linewidths due to the scanner.

Linear Combination (LC) Model software (version 6.2) (Provencher, 2001) was used to perform frequency referencing, eddy current, and baseline correction, and to obtain absolute regional metabolite concentrations and relative metabolite ratios to total creatine (Cr+PCr). A supplied *in vitro* generated basis set of individual metabolite spectra was used to quantify metabolite concentrations in LCModel, where absolute metabolite concentrations of NAA, Glu, total choline to creatine ratios (GPC+PCh/Cr+PCr) and metabolite ratios to Cr+PCr ratios were determined. For partial volume correction and water concentration estimation, each voxel was segmented into gray matter, white matter, and cerebrospinal fluid using Statistical Parametric Mapping (SPM12) software (Penny et al., 2007). Spectra were excluded if they were acquired with a TE greater than 30 ms, or if no water reference data were available, and if spectrum distortions, abnormal lipid peaks, or poor spectra baseline were observed on visual inspection. In addition, spectra with a full width at half length (FWHM) greater than 0.09 ppm and a signal-to-noise ratio (SNR) less than 15 were excluded from our analysis.

### 2.5. Functional neurodevelopmental assessment

Functional neurodevelopmental outcomes were assessed using the Early Learning Outcome Measure (ELOM) tool (Snelling et al., 2019), administered by research assistants at community centers near the TC Newman and Mbekweni clinics. The ELOM tool is a South African school readiness assessment tool used to monitor the population-based development of children aged 50–69 months and is a direct assessment of important milestones related to five developmental domains underpinning the South African early learning curriculum. These domains include gross motor development, fine motor coordination and visual motor integration, emergent numeracy and mathematics, cognition and executive functioning, and emergent literacy and language.

### 2.6. Statistical analysis

Statistical analyses were performed in R (version 4.1.3) with RStudio software (RCoreTeam, 2022). Values less than 1.5 times the interquartile range (IQR) above or below the upper or lower quartiles, respectively, were identified as outliers, and *p*-values less than 0.05 were considered statistically significant. Sociodemographic data were reported as mean (± SD) or frequency (%) for continuous and categorical data, respectively. Continuous data were assessed for normality using the Shapiro–Wilk test and Q-Q plot. For between-group comparisons, normally distributed data were assessed using the t-test, while non-normally distributed data were assessed using the Wilcoxon test, and the chi-squared test was used for categorical data. To compare parietal relative and absolute metabolite concentrations between CHEU and CHU, multiple linear regression analysis was used, correcting for covariates including child sex, child age, and maternal alcohol use during pregnancy. These variables were considered covariates based on previous literature suggesting the influence of these variables on neurometabolic or neuropsychological outcomes in children (Ross and Bluml, 2001; Holmes et al., 2017; Donald et al., 2019). Sociodemographic data with significant between-group differences were also corrected. Associations between regional neurometabolite concentrations and the timing of ART initiation were assessed using the linear regression analysis.

The factorability of the relative MRS data was assessed using Bartlett sphericity and Kaiser–Meyer–Olkin (KMO) tests. Factor analysis was carried out using varimax rotation and a maximum likelihood approach where factors were generated based on metabolite concentrations that were grouped together according to their correlation strengths. Participants who had MRS data available for both parietal gray and white matter and whose data points were not outliers were included in our factor analysis approach. Metabolite concentrations were converted to factor scores using a linear combination weighted by the factor loadings as described by Yiannoutsos et al. (2004). The logistic regression analysis was used to assess whether neurometabolic factors are associated with maternal HIV exposure status, with respective factor scores as predictor variables and HIV exposure as response variables. Variables identified as covariates and corrected for in adjusted linear regression analyses were also corrected for in logistic regression analysis. Pearson’s correlation was used to assess the association between individual neurometabolite concentrations and ELOM outcomes as well as between HIV-associated neurometabolic patterns and ELOM outcomes in CHEU. This analysis included CHEU participants with both neurometabolic and ELOM data.

## 3. Results

### 3.1. Participant demographic characteristics

MRS data were acquired from 152 children enrolled in the DCHS at 6–7 years of age, i.e., 42 CHEU and 110 CHU. Table 1 summarizes the demographic data of the children who had MRS data acquired at 6 years based on HIV exposure. The mean age at scanning was 74.71 months in CHEU and 73.56 months CHU. Mothers living with HIV had similar education, employment status, household income, and alcohol use during pregnancy compared to mothers living without HIV although mothers living with HIV were found to be older with lower rates of depression during pregnancy. Child sex, birth weight, birth length, and head circumference at delivery were similar between groups. Overall, 58% of CHU were exclusively breastfed for at least one month compared to 33% of CHEU. Of the mothers living with HIV, the majority had a lower than detectable viral load limit antenatally although 44% had a CD4 count less than 350 cells per mm^3^ during pregnancy. Due to group differences in maternal age and maternal depression during pregnancy, these variables were included in subsequent adjusted models.

**Table 1:** Sample demographics according to HIV exposure.

	Control (*n* = 110) Mean (SD) or *n* (%)	HEU (*n* = 42) Mean (SD) or *n* (%)	Value of *p*
**Child age at the scan (months)**	73.56 (2.54)	74.71 (2.58)	0.016 *
**Sex**			
Female	54/110 (49.0%)	20/42 (47.6%)	
Male	56/110 (50.9%)	22/42 (52.4%)	0.990
**Maternal education**			0.183
Primary	8/110 (7.2%)	2/42 (4.7%)	
Some secondary	62/110 (56.3%)	17/42 (40.5%)	
Completed secondary	37/110 (33.6%)	20/42 (47.6%)	
Any tertiary	3/110 (2.7%)	3/42 (7.1%)	
**Maternal employment status (employed)**	36/110 (32.7%)	15/42 (35.7%)	0.876
**Maternal relationship status (married or cohabiting)**	44/110 (40%)	22/42 (52.3%)	0.232
**Antenatal household monthly income**			0.300
<R1000–R5000	47/110 (42.7%)	11/42 (26.2%)	
R1000–R5000	47/110 (42.7%)	28/42 (66.7%)	
> R5000	16/110 (14.5%)	3/42 (7.1%)	
**Maternal age at birth (years)**	27.47 (5.92)	27.56 (5.89)	0.029*
**Gestational age at delivery (weeks)**	38.63 (2.13)	38.66 (2.13)	0.757
**Birthweight (g)**	3072.4 (597.1)	3031.2 (628.8)	0.716
**Birth length (cm)**	50.2 (3.4)	48.9 (4.7)	0.115
**Birth head circumference (cm)**	33.63 (2.0)	33.26 (1.96)	0.295
**Maternal alcohol use during pregnancy**	29/110 (26.3%)	6/42 (14.2%)	0.172
**Maternal depression during pregnancy**	35/110 (31.8%)	3/42 (7.1%)	0.003*
**Exclusive breastfeeding for at least 1 month**	64/110 (58.1%)	14/42 (33.33%)	0.010*
**Months of exclusive breastfeeding**	2.4 (1.9)	3.3 (2.1)	0.110
**Maternal antenatal CD4 cell count**			
Median (IQR)(cells/mm^3^)	-	404 (297–618)	
<350 cells/mm^3^	-	13/29 (44%)	
350–500 cells/mm^3^	-	4/29 (13%)	
>500 cells/mm^3^	-	12/29 (41%)	
**Maternal viral load (VL) during pregnancy**			
Median (IQR)(copies/ml)	-	40 (40–41)	
Lower than detectable limit (<40 copies/ml)	-	20/28 (71%)	
VL detectable (>40–1,000 copies/ml)	-	4/28 (14.3%)	
Virally unsuppressed (>1,000 copies/ml)	-	4/28 (14.3%)	
**Antiretroviral drug initiation**			
Before pregnancy	-	18/42 (42.9%)	
During pregnancy	-	24/42 (57.1%)	
**Antiretroviral regimen during pregnancy**			
Monotherapy with AZT	-	4/42 (8.7%)	
2 NRTIs+NNTRI (First line)	-	37/42 (88.1%)	
Second line (PI containing)	-	1/42 (2.4%)	
**Infant prophylaxis**			
NVP (nevirapine) alone	-	36/41 (87.8%)	
NVP+AZT	-	5/41 (12.6%)	

Continuous data were assessed for normality using Shapiro–Wilk test and Q-Q plots. Group demographics were compared using t-tests and Wilcoxon tests for normally and non-normally distributed variables, respectively and X2 tests for categorical data. Missing data: birth length (1 Control, 3 CHEU), maternal antenatal CD4 cell count (13 CHEU), maternal viral load during pregnancy (14 CHEU), Infant prophylaxis (1 CHEU). SD, standard deviation; AZT, zidovine; NRTIs, Nucleoside Reverse Transcriptase Inhibitors; NNTRI, Non-Nucleoside Reverse Transcriptase Inhibitor.*Represents statistically significant values.

**Figure 2:**
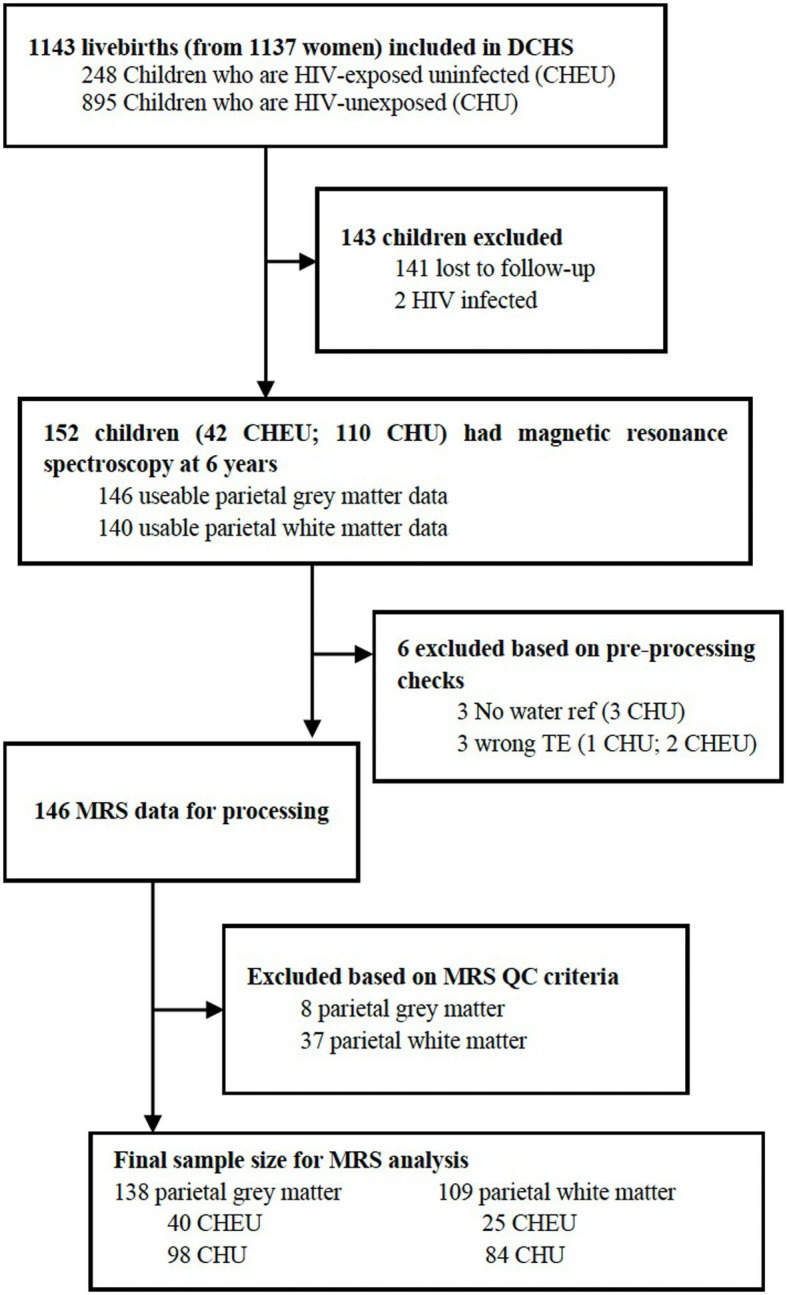
DCHS flow diagram for MRS analysis. DCHS, Drakenstein Child Health Study; ref., reference; TE, time-to-echo; MRS, magnetic resonance spectroscopy; QC, quality control.

Of 152 children who had MRS data acquired at 6–7 years of age, 146 had MRS data available from the parietal gray matter voxel and 140 from the parietal white matter voxel. Prior to MRS data processing, three participants were excluded from the analysis due to no available water reference data, and three participants were excluded due to spectra having a TE that differed from 30 ms. After excluding low-quality spectra based on FWHM and SNR criteria, for parietal gray matter, 138 usable MR spectra (40 CHEU and 98 CHU) and 109 for parietal white matter (25 CHEU and 84 CHU) were included in the analyses. The flow diagram in Figure 2 summarizes the sample flow. Demographic data for the final sample size consisting of 138 participants with useable parietal gray matter spectra and 109 participants with spectra for parietal white matter did not differ significantly from the analysis of the demographic characteristics of the children who had MRS data acquired in Table 1. The tissue composition of the gray matter, white matter, and CSF was not significantly different between groups (Supplementary Table S1).

### 3.2. Differences in parietal neurometabolite levels between CHEU and CHU

**Figure 3:**
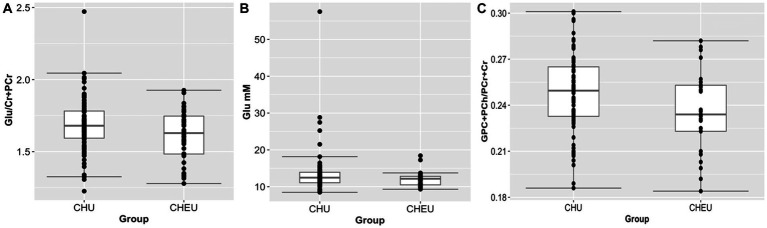
Plots (showing median and IQR) of brain metabolite differences between CHEU and CHU. **(A)** Relative glutamate concentrations (98 CHU, 40 CHEU) in parietal gray matter. **(B)** Absolute glutamate concentrations (98 CHU, 40 CHEU) in parietal gray matter. **(C)** Relative total choline (84 CHU, 25 CHEU) in parietal white matter. Glu, glutamate; Cr+PCr, total creatine (creatine +phosphocreatine) GPC+PCh, total choline (glycerophosphocholine +phosphocholine); CHU, children who are HIV unexposed; CHEU, children who are HIV exposed. Outliers removed from the analysis are data points located outside of the lower and upper limits of the boxplots.

In the parietal gray matter, Glu/Cr+PCr ratios were significantly lower in CHEU when compared to CHU in adjusted analyses (*p* = 0.045; Table 2; Figure 3A). Absolute parietal gray matter Glu concentrations were also found to be significantly lower in CHEU (*p* = 0.035; Table 2; Figure 3B). Neurometabolite findings in the parietal white matter further revealed significant reductions in GPC+PCh/Cr+PCr ratios in CHEU (*p* = 0.039; Table 2; Figure 3C). Both parietal gray matter and parietal white matter findings were significant before and after the removal of outliers and after correcting for maternal age at birth and maternal depression during pregnancy (Supplementary Table S2).

**Table 2:** Adjusted linear regression analysis comparing absolute and relative neurometabolite concentrations in parietal gray matter (40 CHEU, 98 CHU) and parietal white matter (25 CHEU, 84 CHU).

Voxel	Metabolite	*B*	SE	Value of *p*	Metabolite	*B*	SE	Value of *p*
Parietal gray matter	NAA ^a^	−0.02	0.26	0.925	NAA/Cr+PCr	−0.004	0.02	0.831
	Glu ^b^	**−0.64**	**0.32**	**0.046**	Glu/Cr+PCr ^f^	**−0.06**	**0.03**	**0.035**
	Ins ^c^	−0.13	0.13	0.346	Ins/Cr+PCr	−0.01	0.01	0.260
	GPC+PCh^d^	−0.01	0.03	0.549	GPC+PCh/Cr+PCr	−0.003	0.002	0.202
	Cr+PCr ^e^	−0.003	0.13	0.978				
	NAA ^g^	−0.31	1.12	0.781	NAA/Cr+PCr	−0.017	0.025	0.491
	Glu ^h^	3.35	1.95	0.089	Glu/Cr+PCr ^f^	−0.006	0.044	0.892
Parietal white matter	Ins ^i^	−0.35	0.71	0.623	Ins/Cr+PCr	0.005	0.017	0.734
	GPC+PCr ^j^	−0.08	0.14	0.562	GPC+PCh/Cr+PCr	**−0.010**	**0.005**	**0.039**
	Cr+PCr ^k^	0.08	0.98	0.934				

Outliers removed from the analysis: a 5 CHU and 1 CHEU, b 5 CHU and 2 CHEU, c 6 CHU and 7 CHEU, d 5 CHU and 3 CHEU, e 5 CHU and 1 CHEU, f 2 CHU, g 13 CHU and 10 CHEU, h 9 CHU and 6 CHEU, I 11 CHU and 10 CHEU, j 14 CHU and 10 CHEU, k 10 CHU and 9 CHEU. Unstandardised coefficient, B; Standard error, SE; value of *p*, *p*; CHEU, children who are HIV-exposed uninfected; NAA, N-acetyl aspartate; Glu, Glutamate; Ins, myo-inositol; GPC+PCh, total choline (glycerophosphocholine+phosphocholine); Cr+PCr, total creatine (creatine+phosphocreatine); coefficients significant at *p* < 0.05 in bold font. Adjusted for age at scan, sex and maternal alcohol use during pregnancy.

### 3.3. Neurometabolic pattern associated with HIV exposure status

Based on the relative MRS data of 106 CHEU participants, factor analysis generated four neurometabolic patterns in the form of factors (Table 3). Each factor consisted of metabolite factor loadings, where metabolites with the highest factor loadings were considered to contribute strongly to a given metabolite pattern. Each factor was then named according to its most dominant neurometabolites and their location. Factor 1 was dominated by Ins/Cr+PCr in both parietal gray and white matter and was classified as a multi-regional myo-inositol dominated factor. Factor 2 was dominated by equal loadings of Glu/Cr+PCr and Ins/Cr+PCr in the parietal gray matter and was classified as a parietal gray matter glutamate and myo-inositol dominated factor. Dominated by a Glu/Cr+PCr loading in the parietal white matter, factor 3 was classified as a parietal white matter glutamate-dominated factor. Factor 4 was classified as a multi-regional choline-dominated factor, with a strong GPC+PCh/Cr+PCr contribution in both parietal gray and white matter. Subsequent logistic regression findings revealed that factor scores for the parietal gray matter Glu and Ins dominated factor (factor 2) were significantly associated with HIV exposure status in both unadjusted (OR = 0.55, 95% CI 0.17–0.45; *p* = 0.013, Table 4) and adjusted (OR = 0.59; 95% CI 0.35–0.94; *p* = 0.031, Table 4) analyses. This association remained significant after adjusting for maternal age at birth and maternal depression during pregnancy (Supplementary Table S3).

**Table 3:** Factor loadings based on parietal gray matter and parietal white matter relative MRS data from 106 participants (25 CHEU, 81 CHU).

Voxel	Metabolite	Factor loadings			
		1	2	3	4
Parietal gray matter	**NAA/Cr**+**PCr**	0.015	0.273	0.087	−0.052
	**Glu/Cr**+**PCr**	0.084	**0.564**	0.127	0.023
	**Ins/Cr**+**PCr**	**0.764**	**0.555**	−0.288	0.143
	**GPC**+**PCh/Cr**+**PCr**	−0.011	−0.130	−0.041	**0.496**
Parietal white matter	**NAA/Cr**+**PCr**	0.079	0.403	0.366	−0.084
	**Glu/Cr**+**PCr**	0.080	0.297	**0.739**	−0.071
	**Ins/Cr**+**PCr**	**0.958**	−0.015	0.277	−0.009
	**GPC**+**PCh/Cr**+**PCr**	0.063	0.081	−0.047	**0.735**
		Multi-regional myo-inositol dominated factor	Parietal gray matter glutamate and myo-inositol dominated factor	Parietal white matter glutamate dominated factor	Multi-regional choline dominated

Factor loadings in bold font represent the main components of each metabolic pattern. Factors were named according to the functions of the main components of each metabolic pattern. This analysis consists of data from participants who had MRS data available for both parietal gray and white matter and where data points were not outliers. NAA, N-acetyl aspartate; Glu, Glutamate; Ins, myo-inositol; GPC+PCh, total choline (glycerophosphocholine+phosphocholine); Cr+PCr, total creatine (creatine+phosphocreatine).

**Table 4:** Logistic regression analysis of factor scores associations with HIV exposure status.

	Unadjusted logistic regression			*Adjusted logistic regression		
	OR	CI (95%)	*p*	OR	CI (95%)	*p*
Factor 1 Multi-regional myo-inositol dominated factor	1.07	0.19–0.48	0.770	1.03	0.95–1.12	0.530
Factor 2 Parietal gray matter glutamate and myo-inositol dominated factor	**0.55**	**0.17–0.45**	**0.013**	**0.59**	**0.35–0.94**	**0.031**
Factor 3 Parietal white matter glutamate dominated	1.17	0.75–1.83	0.485	1.03	0.94–1.11	0.556
Factor 4 Multi-regional choline dominated	0.73	0.45–1.15	0.181	0.96	0.88–1.04	0.286

Bold print represents statistically significant associations. NAA, N-acetyl aspartate; Glu, Glutamate; Ins, myo-inositol; GPC+PCh, total choline (glycerophosphocholine+phosphocholine); Cr+PCr, total creatine (creatine+phosphocreatine); OR, odds ratio; CI, confidence interval; *p*, value of *p*. *Adjusted for age at scan, sex and maternal alcohol use during pregnancy.

### 3.4. Neurometabolite profiles and motor development correlations in CHEU

**Figure 4:**
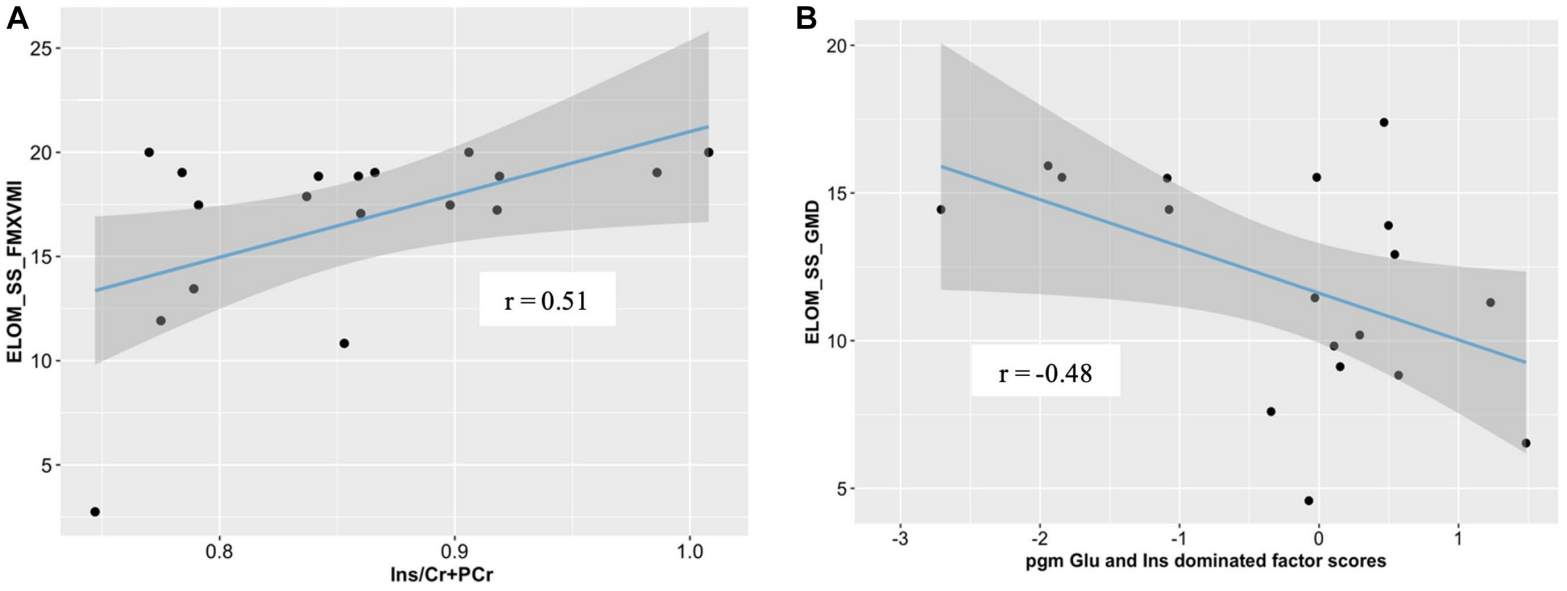
Correlations between neurometabolite profiles and ELOM scores in 18 CHEU. **(A)** Relative parietal white matter Ins concentrations were positively correlated with ELOM scores of Fine Motor Coordination and Visual Motor integration (ELOM_SS_FMXVMI). **(B)** parietal gray matter Glu and Ins dominated factor scores were negatively correlated with ELOM scores of gross motor development (ELOM_SS_GMD). ELOM, Early learning outcome measures; *r*, Pearson’s correlation; parietal gray matter, parietal gray matter; parietal white matter, parietal white matter; Ins, myo-inositol; Cr+PCr, total creatine (creatine +phosphocreatine).

We found parietal white matter Ins/PCr+Cr ratios to be significantly correlated with ELOM scores for fine motor coordination and visual motor integration in CHEU (*r* = 0.51; *p* = 0.032; Figure 4A). Further analyses revealed significant correlations between factor scores of the parietal gray matter Glu and Ins dominated factor, associated with HIV exposure status, and ELOM scores of gross motor development in CHEU (*r* = −0.48, *p* = 0.04, Figure 4B).

### 3.5. Timing of maternal ART initiation and parietal neurometabolite concentrations in CHEU

Adjusted linear regression findings revealed no significant neurometabolite differences between CHEU whose mothers initiated ART prior to pregnancy when compared to ART initiation during pregnancy (Supplementary Table S4).

## 4. Discussion

Our findings implicate alterations to regional neurometabolic profiles in the context of perinatal HIV and/or ART exposure in young school-aged children. In this study, CHEU had reductions in absolute Glu and relative tCho concentrations in the parietal gray matter and parietal white matter, respectively. Furthermore, we found an underlying parietal gray matter neurometabolic pattern in CHEU to be associated with HIV exposure status and found this neurometabolic pattern to be correlated with ELOM scores of gross motor development in CHEU, with significant correlations between parietal white matter Ins concentrations and ELOM scores of fine motor development also observed. This suggests a potential neurometabolic mechanism that may underlie impaired functional neurodevelopmental outcomes in CHEU.

Our findings of regional alterations in Glu concentrations in young school-aged CHEU are consistent with results from previous MRS studies reporting reduced Glu concentrations in the basal ganglia and frontal gray matter of 9-year-old (Robertson et al., 2018) and 11-year-old (Graham et al., 2020) CHEU, respectively. In contrast, in the same cohort, no Glu neurometabolic differences were reported at 7 years of age (Robertson et al., 2018); however, this may be due to larger variability in the data at this age. Furthermore, in our cohort of CHEU at the age of 2 years, elevated Glu/Cr concentrations were reported in the parietal white matter (Bertran-Cobo et al., 2022). One of the key aspects of MRS studies that requires consideration is that of voxel tissue composition. Differences in neurometabolic trends between the gray and white matter have been observed under both normal and pathophysiological conditions (Krukowski et al., 2010) and are expected, especially since these tissues differ in terms of their chemical composition. Contradictory outcomes may therefore also be attributed to age differences with Glu elevations reported in toddlers who are HIV exposed, while reductions are found in school-aged CHEU.

Glu serves as both an excitatory neurotransmitter and an amino acid, playing a crucial role in normal cellular physiology and neurotransmission (Ramadan, 2013). Neuronal Glu is released during synaptic transmission into the extracellular space where it gets taken up by astrocytes and converted into Gln (Meldrum, 2000). Since the concentration of extracellular Glu is much lower than intracellular Glu, the Glu signal detected through MRS is primarily intracellular Glu (Ernst et al., 2010). The dysregulation of the Glu-Gln cycle could result in incomplete Glu recycling contributing to excessive extracellular Glu concentrations that may lead to neurotoxic Glu-mediated excitotoxicity (Vázquez-Santiago et al., 2014). Reduced intracellular Glu, as reported in our study, reflecting excessive extracellular Glu has commonly been implicated in studies on HIV-associated brain injury, and there are several hypotheses for the biological basis and interpretation of this finding: (i) the attenuated ability for HIV-infected astrocytes to take up Glu due to excessive tumor necrosis factor-alpha (TNF-α) release from HIV-infected microglial (Tilleux, 2008), (ii) elevated protein synthesis to repair damage in the brain due to potential exposure to HIV may be contributing to the reduced pool of intracellular Glu (Ernst et al., 2010), and (iii) mitochondrial dysregulation due to certain PMTCT ARV medications (Aldrovandi et al., 2009).

MRS-detected tCho signals consist of a combination of choline-containing compounds predominately found in myelin and neuronal cell membranes and serve as a marker of membrane density; thus, reduced tCho ratios to creatine concentrations in parietal white matter as reported in our study potentially reflect delayed myelination due to *in utero* and early postnatal exposure to HIV (Riley, 2018). An interpretation of reduced tCho concentrations in CHEU as reflecting regional myelin loss in the parietal white matter is broadly supported by findings from previous neuroimaging studies. For example, in 7-year-old CHEU increased fractional anisotropy on DTI was found (Jankiewicz et al., 2017), which is consistent with axonal matrix complexity reductions (Ackermann et al., 2016).

Neurometabolite concentrations of Glu and Ins have both previously been implicated individually in the context of perinatal exposure to HIV (Robertson et al., 2018; Bertran-Cobo et al., 2022). For the first time, we found a neurometabolic pattern implicating both Glu and Ins concentrations. Based on existing scientific literature, neurometabolite patterns dominated either by Glu or Ins have previously been implicated in factor analysis studies on neurochemical signatures in the context of HIV (Lentz et al., 2008). Based on MRS findings from our cohort of CHEU at 2 years of age, a cross-regional parietal matter pattern dominated by Ins across the parietal gray and white matter was found to be associated with HIV exposure status, suggesting a longitudinal signal for neuroinflammation spanning from 2 to 6 years of age (Bertran-Cobo et al., 2022). Given this finding, we hypothesized that at 2 years of age, there is a parietal neurometabolic Ins dominated factor associated with HIV exposure and that as the CHEU gets older, i.e., at the age of 6 years, there is a Glu emergence, resulting in the parietal gray matter Glu and Ins dominated factor as identified in this study.

Given that the parietal lobe is somewhat involved in motor function, including the integration of incoming motor information (Fogassi, 2005), this may explain the associations between neurometabolic profiles and motor development in CHEU. Ins is a marker of neuroglia, primarily those serving as neuroimmune cells. Correlations between parietal white matter Ins ratios to tCr+PCr in CHEU and ELOM scores in fine motor are consistent with a study by Chang et al., where lower regional Ins concentrations in the thalamus were found to be significantly associated with poorer performance in visual motor integration tasks, in children who were exposed to methamphetamine during *in utero* development (Chang, 1995). Furthermore, the association between the HIV-associated parietal gray matter Glu and Ins dominated neurometabolic patterns and gross motor developmental scores suggests that an increase in parietal gray matter Glu and Ins dominated factor scores is associated with lower gross motor developmental scores in CHEU, which have been previously reported. In a recent meta-analysis of neurodevelopmental outcomes (Wedderburn et al., 2022), CHEU had poorer gross motor function when compared to CHU. In addition, this association is further supported by a prior study from the DCHS cohort showing that in CHEU, infant serum inflammatory markers were associated with poorer motor development at the age of 2 years (Sevenoaks et al., 2021).

Findings from our study contribute to a growing body of literature on the regional neurometabolic signatures in children exposed to HIV and/or ART perinatally to the best of our knowledge and are the first to provide some insight into the potential role of parietal neurometabolic profiles in pathophysiology governing impaired functional neurodevelopmental symptomology observed in CHEU. The inclusion of well-characterized participants from a sub-Saharan African LMIC setting with a rich body of clinical, socioeconomic, and demographic information available and the fact that covariates were considered in our analyses are some strengths of this study. We recognize that our study had some limitations which could have affected our findings and thus our interpretation thereof. First, we had a small sample size and thus reduced statistical power, especially in the correlation analyses. Second, neurometabolite concentrations were only quantified from voxels located in particular regions in the parietal matter, thus limiting the brain metabolite findings to this region of interest. Third, despite taking covariates into account, we recognize that there may be underlying covariates not corrected in our analysis that could have influenced our findings.

Future research should be undertaken to investigate these neurometabolic profiles in other brain regions, such as frontal and limbic regions. Furthermore, further research focusing on investigating neurochemical outcomes in CHEU as adolescents or young adults is warranted, where neurometabolic findings could perhaps be investigated in relation to neurological outcomes relevant to individuals emerging into adulthood. Given that gross motor development in CHEU was associated with an neurometabolic pattern in this study and inflammatory serum markers in another study (Sevenoaks et al. 2021), a future study could perhaps focus on exploring the link between regional neurometabolites and peripheral or central inflammatory markers in CHEU. In addition, future research could also be geared toward pre-clinical animal studies exploring the exact mechanism by which tCho and Glu neurometabolite alterations may be occurring in CHEU to gain a better understanding of the pathophysiology governing neurochemical outcomes in CHEU. This could also be useful in the development of therapeutic interventions aimed at improving functional neurodevelopmental outcomes in children who are exposed to HIV and/or ART during *in utero* and/or early development.

In conclusion, by assessing individual relative and absolute neurometabolite concentrations in CHEU and CHEU, significant brain neurometabolite alterations were observed in both parietal gray matter and parietal white matter in this cohort study of CHEU. Using an exploratory factor analysis approach, underlying regional brain metabolite patterns were identified, with an intra-regional neurometabolic pattern found to be significantly associated with HIV exposure status. Furthermore, a parietal gray matter Glu and Ins dominated factor as well as parietal white matter Ins/tCr+PCr ratios were significantly correlated with motor development in CHEU. Findings from this study support the notion that neurometabolic alterations are prevalent in the context of exposure to maternal HIV/ART perinatally and highlight a potential pathophysiological brain neurometabolic mechanism that may be contributing to motor developmental impairment described in CHEU.

## Data availability statement

The original contributions presented in the study are included in the article/Supplementary material, further inquiries can be directed to the corresponding author.

## Ethics statement

The studies involving human participants were reviewed and approved by the Western Cape Provincial Health Research Committee and Faculty of Health Science, Human Research Ethics Committee at the University of Cape Town. Written informed consent to participate in this study was provided by the participants’ legal guardian/next of kin.

## Author contributions

SW: data collection, methodology, gathering literature for the manuscript, formal analysis and interpretation, visualization, drafting the original manuscript, and review and editing. FR: methodology, supervision, and writing—review and editing. CW contributions to analysis, interpretation, and writing—review and editing. JR, LB, and CN: data collection, methodology, and writing—review and editing. NH and SJ: methodology and writing—review and editing. HZ: conceptualization, methodology, resources, and writing—review and editing. DS: supervision, conceptualization, methodology, resources, and writing—review and editing. KD: conceptualization, methodology, investigation, resources, supervision, and writing—review and editing. All authors contributed to the article and approved the submitted version.
