# Supplementary material for: 1H-MRS neurometabolite profiles and motor development in school-aged children who are HIV-exposed uninfected: a birth cohort study
Source: Front Neurosci. 2023 Oct 12;17:1251575. doi: 10.3389/fnins.2023.1251575 (PMC10600451; doi:10.3389/fnins.2023.1251575)
Supplement: Supplementary file 1 [file Table_1.DOCX]

**Supplementary Results**

| Voxel | Group | n | %GM | % WM | % CSF |
| --- | --- | --- | --- | --- | --- |
| pgm | CHU | 98 | **76.3 (0.02)** | 16.5 (0.02) | 7.1 (0.02) |
|  | CHEU | 40 | **76.9 (0.03)** | 15.8 (0.02) | 7.1 (0.02) |
| pwm | CHU | 84 | 46.1 (0.06) | **50.9 (0.06)** | 0.02 (0.01) |
|  | CHEU | 25 | 47.1 (0.06) | **49.9 (0.06)** | 0.03 (0.01) |

**Supplementary Table 1**

Average brain tissue composition in the parietal grey and white matter voxels according to HIV exposure status

Data is displayed as mean (SD) percentages. Percentages printed in bold font indicate the tissue of interest in respective voxels. n, number of participants; GM, grey matter; WM, white matter; CSF, cerebrospinal fluid; CHEU, children who are HIV-exposed uninfected; CHU, children who are HIV unexposed.

|  |  | | | | | |  |
| --- | --- | --- | --- | --- | --- | --- | --- |
| Metabolite | *Adjusted for maternal age at birth | | | **Adjusted for maternal depression during pregnancy | | |  |
|  | B | SE | p | B | SE | p |  |
| Pgm Glu^a^ | -0.70 | 0.32 | 0.030 | -0.71 | 0.33 | 0.032 |  |
| Pgm Glu / PCr +Cr^b^ | -0.07 | 0.03 | 0.034 | -0.06 | 0.03 | 0.048 |  |
| Pwm GPC+PCh / PCr +Cr | -0.010 | 0.01 | 0.031 | -0.01 | 0.01 | 0.0352 |  |

**Supplementary Table 2**

Linear regression analysis adjusting for maternal age at birth and maternal depression during pregnancy comparing neurometabolite concentrations that had significant between-group differences, in pgm and pwm.

Outliers removed from the analysis: ^a^ 5 CHU and 2 CHEU, ^b^ 2 CHU; unstandardised coefficient, B; Standard error, SE; p-va1ue, p; CHEU, children who are HIV-exposed uninfected; Glu, Glutamate; GPC+PCh, total choline (glycerophosphocholine +phosphocholine); Cr+PCr, total creatine (creatine +phosphocreatine). *Adjusted for age at scan, sex, maternal alcohol use during pregnancy and maternal age at birth. ** Adjusted for age at scan, sex, maternal alcohol use during pregnancy and maternal depression during pregnancy.

**Supplementary Table 3**

Logistic regression analysis of factor scores associated with HIV exposure status, adjusted for maternal age at birth and maternal depression during pregnancy.

|  | |  | | |  | | |
| --- | --- | --- | --- | --- | --- | --- | --- |
|  | *Adjusting for maternal age at birth | | | **Adjusting for maternal depression during pregnancy | | |  |
|  | | OR | CI  (95%) | p | OR | CI  (95%) | p |
| **Factor 1**  Multi-regional myo-inositol dominated factor | | 1.027 | 0.95 - 1.12 | 0.529 | 1.024 | 0.94 - 1.11 | 0.571 |
| **Factor 2**  Pgm glutamate and myo-inositol dominated factor | | **0.910** | **0.84 - 0.99** | **0.030** | **0.903** | **0.83- 0.98** | **0.019** |
| **Factor 3**  Pwm glutamate dominated | | 1.025 | 0.94 - 1.11 | 0.485 | 1.014 | 0.93- 1.10 | 0.730 |
| **Factor 4**  Multi-regional choline dominated | | 0.956 | 0.88 - 1.04 | 0.286 | 0.949 | 0.87 - 1.03 | 0.216 |

Bold print represents statistically significant associations. OR, odds ratio; CI, confidence interval; p, p-value . *Adjusted for age at scan, sex, maternal alcohol use during pregnancy and maternal age at birth. ** Adjusted for age at scan, sex, maternal alcohol use during pregnancy and maternal depression during pregnancy.

Outliers removed from the analysis: ^a^ 2 CHEU. Unstandardised coefficient, B; Standard error, SE; p, p-va1ue; n, sample size. CHEU, children who are HIV-exposed; Glu, Glutamate; GPC+PCh, total choline (glycerophosphocholine +phosphocholine); Cr+PCr, total creatine (creatine +phosphocreatine); pgm, parietal grey matter; pwm, parietal white matter; ARV, antiretroviral. Brain metabolites previously shown to be significantly different between groups were used in this analysis.

**Supplementary Table 4**

Adjusted linear regression analysis comparing neurometabolite concentrations between mothers who initiated antiretroviral treatment prior to pregnancy and mothers who initiated antiretroviral treatment during pregnancy.

| Voxel | n | Neurometabolite | B | SE | p |
| --- | --- | --- | --- | --- | --- |
| pgm | 17 ART initiation before pregnancy  23 ART initiation during pregnancy | pgm absolute Glu^a^ | -1.20 | 0.81 | 0.146 |
|  |  | pgm relative Glu (Glu/Cr+PCr) | -0.02 | 0.06 | 0.782 |
|  |  |  | | | |
| pwm | 15 ART initiation before pregnancy  10 ART initiation during pregnancy | pwm relative GPC+PCh (GPC+PCh/Cr+PCr) | -0.005 | 0.01 | 0.298 |
